# Outcomes of the national programme on prevention of mother-to-child transmission of hepatitis B virus in China, 2016–2017

**DOI:** 10.1186/s40249-019-0576-y

**Published:** 2019-08-05

**Authors:** Ya-Ping Qiao, Min Su, Yao Song, Xiao-Yan Wang, Zhen Li, Yan-Lin Li, Li-Xia Dou, Qian Wang, Katrina Hann, Guo-Min Zhang, Xiao-Na Huang, Yu-Ning Yang, Xi Jin, Ai-Ling Wang

**Affiliations:** 1 National Centre for Women and Children’s Health, China CDC, Beijing, China; 2 Community Health Service Centre, Dingzigu Street, Hongqiao District, Tianjin, China; 3 Chengdu University of Traditional Chinese Medicine, Chengdu, China; 4 Sustainable Health Systems, Freetown, Sierra Leone; 5 Institute of Immunization, China CDC, Beijing, China; 6 UNICEF China, Beijing, China

**Keywords:** Perinatal, Birth dose, Immunization, elimination, Vaccination guideline

## Abstract

**Background:**

In addition to providing free hepatitis B vaccine (HBvacc) series to all infants in China since 2005, the national programme on prevention of mother-to-child transmission (PMTCT) of hepatitis B virus (HBV) started providing free hepatitis B immunoglobulin for all new-borns born to hepatitis B surface-antigen (HBsAg) positive mothers in 2010. However, few studies have evaluated the effectiveness of the PMTCT programme. Therefore, we aimed to investigate the outcomes of the programme and identify associated factors.

**Method:**

Using a cross-sectional study design, we collected data on 4112 pairs of HBsAg-positive mothers and their children aged 7–22 months in four representative provinces through interviews and medical record review. We tested HBsAg and hepatitis B surface antibody (anti-HBs) of children by enzyme-linked immunosorbent assay at designated maternal and child hospital laboratories. We used logistic regression to analyse factors associated with child HBsAg and anti-HBs positivity.

**Results:**

Thirty-five children were HBsAg positive, indicating the mother-to-child transmission (MTCT) rate was 0.9% (0.6–1.1%). The anti-HBs positive rate was 96.8% (96.3-97.4%). Children receiving HBvacc between 12 and 24 h of birth were 2.9 times more likely to be infected than those vaccinated in less than 12 h (adjusted odds ratio [a*OR*] = 2.9, 95% confidence interval [*CI*]: 1.4–6.3, *P* = 0.01). Maternal hepatitis B e-antigen (HBeAg) positivity was associated with higher MTCT rate (a*OR* = 79.1, 95% *CI*: 10.8–580.2, *P* <  0.001) and lower anti-HBs positive rate (a*OR* = 0.4, 95% *CI*: 0.3–0.6, *P* <  0.001). Children with low birth weight (LBW) were 60% less likely to be anti-HBs positive than those with normal birth weight (a*OR* = 0.4, 95% *CI*: 0.2–0.8, *P* = 0.01).

**Conclusions:**

The MTCT rate was lower than the 2030 WHO elimination goal, which implies the programme is on track to achieve this target. As earlier HBvacc birth dose (HBvcc-BD) was associated with lower MTCT rate, we suggest that the PMTCT programme work with the Expanded Programme on Immunization (EPI) to modify the current recommendation for early HBvcc-BD to a requirement. Our finding that LBW was associated with lower anti-HBs positivity points to the need for further studies to understand factors associated with these risks and opportunities for program strengthening. The programme needs to ensure providing essential test to identify HBeAg-positive mothers and their infants and provide them with appropriate medical care and follow-up.

**Electronic supplementary material:**

The online version of this article (10.1186/s40249-019-0576-y) contains supplementary material, which is available to authorized users.

## Multilingual abstracts

Please see Additional file 1 for translations of the abstract into the five official working languages of the United Nations.

## Background

Hepatitis B is a global public health concern with 257 million people living with hepatitis B virus (HBV) infection (hepatitis B surface-antigen [HBsAg] positive) worldwide [1]. The Western Pacific region accounted for almost 45% of the total number of 257 million globally [1]. Without intervention, the mother-to-child transmission (MTCT) risk in Asia for infants born to mothers with only HBsAg positivity and with both HBsAg and hepatitis B e-antigen (HBeAg) positivity is high, 5–30% and 70–100% respectively [1]. Globally, the proportion of children under 5 years who become chronically infected has dramatically declined from 4.7% in the pre-vaccine era (prior to the 1980s) to 1.3% in 2015 because of the expansion of routine hepatitis B vaccination [1]. The World Health Organization (WHO) has subsequently set a global target to achieve a 0.1% prevalence of HBV infection in children by 2030, with associated objectives to prevent MTCT through expanding the coverage of Hepatitis B vaccine birth dose (HBvacc-BD) to 90% of infants [2–4].

It is estimated that more than 90 million persons are chronically infected with HBV in China [5]. Annually about 100 000 persons are newly infected with HBV, among which 40–50% are infected through vertical transmission [6]. The Chinese Central Government started to provide free three-dose hepatitis B vaccines (HBvacc) for all infants through the nation-wide Expanded Programme on Immunization (EPI) in 2005, guided by the National Immunization Schedule [7, 8]. These efforts have resulted in reductions in HBsAg-positive prevalence in both the general population (9.2 to 7.2% from 1992 to 2006) and children under 5 years (0.96 to 0.32% from 2006 to 2014) [7].

While the results of EPI are laudable, hepatitis transmission still persists. The MTCT rate among HBV-exposed infants ranges from 3 to 5% [9, 10], with approximately 50 000 infants infected annually in China [7, 11]. In 2013, the HBsAg prevalence among pregnant women was 6% [12]. Hence, there were about 1 million such deliveries annually at risk of MTCT. Among those deliveries, 30% of infants are born to HBsAg- and HBeAg-positive mothers with high MTCT risk [5].

As HBV MTCT remains an important public health concern in China, the national programme on prevention of mother-to-child transmission (PMTCT) of human immunodeficiency virus (HIV) integrated syphilis and HBV into its mandate in 2010. The programme, which covers all women accessing antenatal care services across the country, now tests all women for HBsAg during pregnancy and administers hepatitis B immunoglobulin (HBIG) and HBvacc-BD for all HBV-exposed new-borns within 24 h of birth free-of-charge [13]. In addition, China endorsed the 2018–2030 Asia Pacific Regional Framework for Triple Elimination of MTCT of HIV, syphilis and HBV in 2017 [14]. However, despite these important programmatic and policy actions, the outcomes of HBV component of the PMTCT programme have not yet been systematically evaluated. Identifying the current HBV MTCT rate will allow for projections and target setting in relation to the global and regional elimination goals. In addition, understanding factors associated with programmatic outcomes is essential to better understand how to make policy and programmatic recommendations that ensure services reach the ‘last mile’ of mother-infant pairs. Therefore, our study aimed to investigate the outcomes and associated factors of the HBV component of the national PMTCT programme in China between 2016 and 2017.

## Methods

### Study design

We conducted a cross-sectional study in four representative provinces of China between October 2017 and January 2018. We chose Guangdong, Zhejiang, Shaanxi, and Hebei province representing different levels of the prevalence of HBsAg positivity among pregnant women in China as well as representation across the regions. Among those four provinces, Zhejiang and Guangdong located in eastern China, Hebei located in central China, and Shaanxi located in western China. We selected two to five counties per province based on the highest estimated number of HBV-exposed infants to reach to ensure that we included a substantial number of mother-child pairs. We applied a two-prong approach to collect the data. The study team used a structured-questionnaire to collect maternal demographics, laboratory test to identify serological outcomes of HBV-exposed children, and reviewed the medical records of the mother-child pairs and immunization records of the children to extract data on maternal laboratory test results, delivery, and immunoprophylactic management of children. In addition, we reported this study following the STROBE guidelines [15].

### General setting

The national programme on PMTCT of HIV, syphilis, and HBV, funded by the Chinese Central Government, started in 2010 in 41% (1156 of 2851) of counties and districts and expanded nation-wide in 2015. In addition to preventing vertical transmission of HIV and syphilis, the programme aims to reduce the HBV MTCT rate through screening of pregnant women and timely HBIG provision for HBV-exposed new-borns. The PMTCT programme works closely with the EPI to reinforce effective coverage of the three-dose Hepatitis B vaccination schedule for HBV-exposed infants (see Table 1 for details of the programme intervention components and associated indicators). According to the national action plan [13] and the 2005 Standard for Vaccination Work Specification [16], prophylaxis for infants born to HBsAg-positive women consists of HBvacc and HBIG administered within 24 h of birth, followed by completion of the vaccine series. In line with WHO recommendations, the HBvacc-BD is required within 24 h of birth, but strongly recommends HBV-exposed infants receive it as soon as possible following birth [8].

**Table 1:** The intervention components, program outputs and outcomes, and associated indicators for the PMTCT of HBV component of the national programme on PMTCT of HIV, syphilis, and HBV in China

Intervention component	Program output	Indicator	Program outcome	Indicator
Pregnant Women				
HBV screening of pregnant women attending antenatal care	Screening of pregnant women for HBsAg	Coverage of HBV screening for pregnant women		
HBV-exposed Infants				
HBIG 100 IU within 24 h of birth	Timely birth-dose (within 24 h of birth) of HBIG	Timely HBIG coverage (proportion of HBV-exposed infants receiving timely birth-dose of HBIG)	Reduction in HBV MTCT	HBsAg positivity amongst HBV-exposed infants in 1 year (MTCT rate)
Three-dose HBvacc (provided by EPI)	Timely HBvacc-BD (within 24 h of birth)	Timely HBvacc-BD coverage (proportion of HBV-exposed infants receiving timely HBvacc-BD)	Sero-protection from immunization	Anti-HBs positivity amongst HBV-exposed infants within 7–12 months (sero-protection rate)
	Completion of the second and third dose HBvacc (after 1 month and 6 month of birth)	Proportion HBV-exposed infants completing three-dose HBvacc series		

*PMTCT* prevention of mother-to-child transmission, *HBV* hepatitis B virus, *HBsAg* hepatitis B surface antigen, *HBIG* hepatitis B immunoglobulin, *IU* international unit, *HBvacc-BD* hepatitis B vaccine birth dose, *MTCT* mother-to-child transmission, *HBvacc* hepatitis B vaccine, *EPI* expanded programme on immunization, *anti-HBs* hepatitis B surface antibody

### Study population

We defined inclusion criteria for HBsAg-positive mothers as those with the following attributes in the hospital information management system: (1) screened as HBsAg-positive by laboratory test; and (2) health care facility-delivery at a study site between April 2016 and March 2017. We excluded mothers who were: (1) ill at the time of the study contact; and/or (2) not contactable following requirements of study recruitment protocol. We defined inclusion criteria for HBV-exposed infants as: (1) born to HBsAg-positive mothers who met the inclusion criteria for the study; (2) born in health facilities of study sites between April 2016 and March 2017; (3) having completed the three-dose HBV vaccination schedule; (4) alive at time of study. We excluded infants who were: (1) ill at the time of the study contact; (2) not contactable following requirement of study recruitment protocol; and/or (3) guardian refusal for blood draw and testing procedure.

We applied the formula for the cross-sectional observational study to calculate the sample size:

$$ n=\frac{Z_{\alpha}^2\times p\times \left(1-p\right)}{{\left(\delta p\right)}^2} $$

According to recent research in China, the reported HBsAg positivity rate among HBV-exposed infants after immunization intervention is 3–5% [9, 10]. We utilized a mean HBsAg positivity rate of 4%. With an allowable deviation of 15%, we calculated an approximate sample size of 4182 mother-child pairs.

First, we chose four provinces by stratified sampling. We stratified the 31 provinces, autonomous regions, and municipalities (not including Macau, Hong Kong, and Taiwan) in China served by the programme into quartiles of prevalence of HBsAg positivity among pregnant women in 2016. In each stratum, we used simple random sampling to select one province. We used convenience sampling to select between two to five counties/districts per province based on the highest estimated number of HBV-exposed infants to reach at least *n* = 1100 in each province. In a total, we chose 13 counties/districts from Guangdong, Zhejiang, Shaanxi, and Hebei provinces as the research sites. In the study sites, we recruited all mother-child pairs identified through the hospital information system at all health care facilities offering delivery services.

### Data collection and variables

After obtaining informed consent and parental assent from the HBsAg-positive mothers, the study team interviewed mothers using a structured questionnaire to collect maternal demographics. The study team collected serological outcomes of HBV-exposed children by collecting blood samples and sending them for laboratory tests at designated maternal and children hospitals to detect HBsAg and hepatitis B surface antibody (anti-HBs) markers by enzyme-linked immunosorbent assay (ELISA). The detection limits for anti-HBs and HBsAg were 10 IU/L and less than 1 IU/ml respectively. The study team reviewed the medical records of the mother-child pairs and immunization records of the children to extract data on maternal laboratory test results, mode of delivery and level of delivery hospitals; and child gestational age and birth weight and immunoprophylactic management through medical records.

We defined timely HBIG and HBvacc-BD coverage as the proportion of children who received HBIG and HBvacc-BD within 24 h of birth, respectively. We defined MTCT rate as the proportion of HBsAg positive and anti-HBs negative children among those born to HBV-positive mothers; and sero-protection rate (measured between 1 and 18 months from the third-dose HBvacc) as the proportion of HBsAg negative and anti-HBs positive children among those born to HBV-positive mothers. We defined short testing interval as less than 7 months between time of receiving the third-dose HBvacc and the time of the serological test study procedure. We defined low birth weight (LBW) as less than 2500 g.

We classified HBvacc type into three groups: (1) 10 μg yeast vaccine, derived from *Saccharomyces cerevisiae*, 10 μg/0.5 ml, produced by Beijing Tiantan Biological Products Co., Ltd., Beijing, China, or Shenzhen Kangtai Biological Products Co., Ltd., Shenzhen, China, and derived from *Hansenula ploymorpha*, 10 μg/0.5 ml, produced by Aimei Hissen Vaccine (Dalian) Co., Ltd., Dalian, China; (2) 20 μg CHO vaccine, derived from Chinese hamster ovary cells (CHO), 20 μg/1 ml, produced by North China Pharmaceutical, Jintan biological products Co., Ltd., Shijiazhuang, China; 3) 10 μg CHO vaccine, derived from CHO, 10 μg/0.5 ml, produced by North China Pharmaceutical, Jintan biological products Co., Ltd., Shijiazhuang, China.

### Statistical analysis

Prior to data entry, we reviewed the questionnaire data and undertook checks for quality assurance. We double entered into and validated data using Epidata software (version 3.1, The EpiData Association, Odense, Denmark). We analysed the data using SPSS software (version 23.0, IBM Corp, Armonk, NY, USA). We presented categorized variables of demographic, immunization, birth history, and laboratory characteristics using frequencies and proportions, and continuous variables with normal distribution with mean and standard deviation (*SD*). We summarized programme indicators using frequencies, proportions, and 95% confidence intervals. We analysed the factors associated each of the MTCT and sero-protection rates using Pearson’s chi-squared or Fisher’s exact test, as appropriate. We entered the factors with a *P* value of < 0.1 into a binary logistic regression to build a final model for each of the MTCT and sero-protection rates. We consider a *P* value of < 0.05 as statistically significant.

## Results

### Characteristics of the study population

**Fig. 1:**
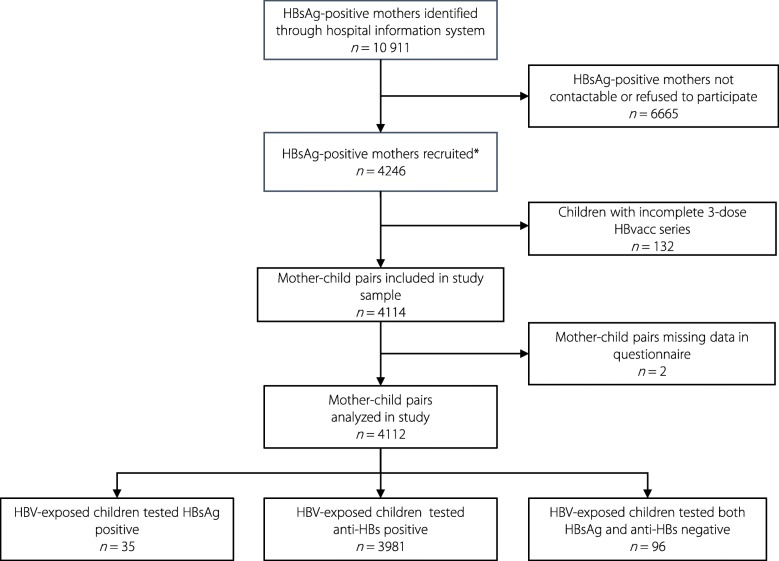
The recruitment process of mother-infant pairs in the study of outcomes of the national programme on PMTCT of HBV in China, 2016–2017. PMTCT: Prevention of mother-to-child transmission; HBV: Hepatitis B virus; HBsAg: Hepatitis B surface antigen; HBeAg: Hepatitis B e antigen; anti-HBs: Hepatitis B surface antibody; HBvacc: Hepatitis B vaccine; 3-dose HBvacc series: First dose within 24 hours of birth, second dose after 1 month, third dose after 6 months, *Children had HBV serological marker test

Through the recruiting process, we analysed data from 4112 mother-child pairs (Fig. 1). The average maternal age was 31 ± 5 years (range: 18–45 years). The majority of mothers had a high middle school education or above (2730, 66%), were from rural areas (2818, 69%), and had given birth at least once previously (2380, 58%). About 30% (1221) of mothers were HBeAg positive. In total, 26% (1047) of mothers had an HBV DNA test during pregnancy documented, of which 291 resulted in HBV DNA level higher than 2 000 000 UI per milliliter. About 9.5% (392) of mothers had taken antiviral drugs during the pregnancy. The average age of children was 14 ± 4 months (range: 7–22 months) (Table 2).

**Table 2:** Characteristics of HBsAg-positive mothers and their HBV-exposed children in four provinces, China 2016–2017

Characteristics	*N*	%
Total	4112	100.0
Province		
Guangdong	1297	31.5
Hebei	920	22.4
Shaanxi	718	17.5
Zhejiang	1177	28.6
Maternal age (years)		
18–24	280	6.8
25–34	2992	72.8
35–45	840	20.4
Maternal educational level		
Junior middle school and below	1382	33.6
High middle school and above	2730	66.4
Maternal residence rural/urban		
Rural	2818	68.5
Urban	1294	31.5
Maternal Parity		
1	1732	42.1
> 1	2380	57.9
Maternal HBeAg status during this pregnancy		
Positive	1221	29.7
Negative	2740	66.6
Unknown	151	3.7
Maternal HBV DNA during this pregnancy (IU/ml)		
< 100	182	4.4
100–2 000 000	574	14.0
> 2 000 000	291	7.1
Unknown	3065	74.5
Maternal antiviral treatment during this pregnancy		
Yes	392	9.5
No	3720	90.5
Level of delivery hospital		
County level and below	2594	63.1
Prefectural and above	1518	36.9
Mode of delivery		
Vaginal delivery	2349	57.1
Caesarean section	1763	42.9
Child age (months)		
7–12	1626	63.1
13–22	2486	36.9
Child gender		
Male	2185	53.1
Female	1927	46.9
Gestational age (weeks)		
< 37	236	5.7
37–43	3876	94.3
Child birth weight (g)		
< 2500 (LBW)	131	3.2
2500–5400	3981	96.8
Interval between birth and HBvacc-BD (hours)		
< 12	3579	87.0
12–24	466	11.3
> 24	67	1.7
Type of HBvacc-BD		
10 μg yeast vaccine	3637	88.5
20 μg CHO vaccine	285	6.9
10 μg CHO vaccine	190	4.6
Interval between birth and HBIG dose (hours)		
< 12	3888	94.6
12–24	182	4.4
> 24	22	0.5
No	20	0.5
Received both HBvacc birth dose and HBIG within 12 h of birth		
Yes	3538	86.0
No	574	14.0
Interval between third dose HBvacc and HBV serological test (months)		
< 7	1666	40.5
7–18	2446	59.5

*HBsAg* hepatitis B surface antigen, *HBV* hepatitis B virus, *HBeAg* hepatitis B e antigen, *HBvacc* hepatitis B vaccine, *HBvacc-BD* hepatitis B vaccine birth dose, *HBIG* hepatitis B immunoglobulin, *LBW* low birth weight, *CHO* Chinese hamster ovary, *IU* international unit

### Programme outcomes

During 2016–2017, the programme achieved timely HBIG and HBvacc-BD coverage of 99% (4070/4112) and 98% (4045/4112) respectively. Children who received yeast vaccine at birth accounted for 88% (3637) of the sample, and 86% (3538) were administered both HBvacc-BD and HBIG within 12 h of birth. The programme achieved an MTCT rate of 0.9% (0.6–1.1%), with 35 children tested HBsAg positive, and a sero-protection rate of 96.8% (96.3-97.4%), with 3981 children tested anti-HBs positive. Out of 35 HBsAg-positive children, 94% were born to HBsAg- and HBeAg-positive mothers during this pregnancy. Of 4112 children, serological test results for 96 (2.3%) were both HBsAg and anti-HBs negative (Table 2).

### Univariate and multivariate analysis on MTCT rate

The timing of HBvacc-BD was associated with MTCT. For children administered the HBvacc-BD between 12 and 24 h in birth, the adjusted odds of MTCT was 1.9 times higher than that of children immunized within 12 h of birth (2.4% vs 0.6%, adjusted odds ratio [a*OR*] = 2.9, 95% confidence interval [*CI*]:1.4–6.3, *P* = 0.01). The MTCT rate was higher among children born to HBeAg positive mothers than those born to HBeAg negative mothers (2.7% vs 0.04%, a*OR* = 79.1, 95% *CI*: 10.8–580.2, *P* <  0.001). We found a higher MTCT rate among children delivered in hospitals at county level or below than in those delivered at prefectural hospitals or above (1.1% vs 0.4%, a*OR* = 3.3, 95% *CI*: 1.3–8.0, *P* = 0.01). We found no significant association between MTCT and maternal age, educational level, parity, or antiviral treatment during this pregnancy, or with the child’s gender, gestational age, HBIG administration, or birth weight (Table 3).

**Table 3:** Univariate and multivariate analysis of factors associated with HBV infection among children born to HBsAg-positive mothers in four provinces, China 2016–2017

Characteristics	Number of observations	HBsAg Positive	Univariate analysis		Multivariable analysis	
		*N*(%)	*OR*(*95% CI*)	*P*-value	a*OR*(*95% CI*)	*P*-value
Maternal age (years)						
18–24	280	3(1.1)	2.3 (0.5–10.2)	0.2^#^		
25–34	2992	28(0.9)	2.0 (0.7–5.6)			
35–45	840	4(0.5)	1			
Maternal educational level						
Junior middle school and below	1382	12(0.9)	1.0 (0.5–2.1)	0.9		
High middle school and above	2730	23(0.8)	1			
Maternal residence rural / urban						
Rural	2818	28(1.0)	1.8 (0.8–4.2)	0.1		
Urban	1294	7(0.5)	1			
Maternal parity						
1	1732	18(1.0)	1.5 (0.8–2.8)	1.3		
> 1	2380	17(0.7)	1			
Maternal HBeAg status during this pregnancy						
Positive	1221	33(2.7)	76.1 (10.4–556.9)	< 0.001^#^	79.1 (10.8–580.2)	< 0.001
Negative	2740	1(0.04)	1		1	
Unknown	151	1(0.7)	18.3 (1.1–293.4)		16.7 (1.0–269.6)	0.05
Maternal antiviral treatment during this pregnancy						
Yes	392	1(0.3)	0.3 (0.0–2.0)	0.25*		
No	3720	34(0.9)	1			
Level of delivery hospital						
County level and below	2594	29(1.1)	2.8 (1.2–6.9)	0.02	3.3 (1.3–8.0)	0.01
Prefectural and above	1518	6(0.4)	1		1	
Mode of delivery						
Vaginal delivery	2349	23(1.0)	1.4 (0.72–2.9)	0.3		
Caesarean section	1763	12(0.7)	1			
Child gender						
Male	2185	17(0.8)	0.8 (0.4–1.6)	0.6		
Female	1927	18(0.9)	1			
Gestational age (weeks)						
< 37	236	3(1.3)	1.5 (0.5–5.1)	0.5*		
37–43	3876	32(0.8)				
Child birth weight (g)						
< 2500 (LBW)	131	3(2.3)	2.9 (0.9–9.6)	0.09*	2.7 (0.8–9.7)	0.1
2500–5400	3981	32(0.8)	1		1	
Type of HBvacc–BD						
10 μg yeast vaccine	3637	29(0.8)	0.8 (0.2–3.2)	0.5		
20 μg CHO vaccine	285	4(1.4)	1.3 (0.2–7.4)			
10 μg CHO vaccine	190	2(1.1)	1			
Interval between birth and HBvac-BD (hours)						
Within 12	3579	23(0.6)	1	0.001^#^	1	
12–24	466	11(2.4)	3.7 (1.8–7.7)		2.9 (1.4–6.3)	0.01
> 24	67	1(1.5)	2.4 (0.3–17.6)		2.1 (0.3–17.7)	0.5
Administered HBIG						
Yes	4092	35(0.9)	0.99 (0.98–0.99)	1.0*		
No	20	0(0.0)	1			
Interval between 3rd dose HBvacc and HBV serological marker test (months)						
< 7	1666	12(0.7)	0.8 (0.4–1.5)	0.5		
7–18	2446	23(0.9)	1			

*HBV* hepatitis B virus, *HBsAg* hepatitis B surface antigen, *HBeAg* HBV e antigen, *HBvacc* hepatitis B vaccine, *HBvacc-BD* hepatitis B vaccine birth dose, *HBIG* hepatitis B immunoglobulin, *LBW* low birth weight, *CHO* Chinese hamster ovary, #: trend, *: Fisher’s exact test, *OR* odds ratio, a*OR* adjusted odds ratio, factors significant at *P* <  0.1 in univariate analysis entered into the regression model)

### Univariate and multivariate analysis on sero-protection rate

We found a lower sero-protection rate among children with LWB than those born over 2500 g (93% vs 97%, a*OR* = 0.4, 95% *CI*: 0.2–0.8, *P* = 0.01). Children with a shorter testing interval showed a higher sero-protection rate than those with a longer testing interval (98% vs 96%, a*OR* = 2.2, 95% *CI*: 1.5–3.4, *P* <  0.001). The proportions of anti-HBs negative children among HBV-exposed children born to HBeAg positive and HBeAg negative mothers were 2.2% (59/2740) and 5.4% (66/1221) respectively (*P* <  0.001) (Table 4).

**Table 4:** Univariate and multivariate analysis of factors associated with sero-protection among children born to HBsAg-positive mothers in four provinces, China 2016–2017

Characteristics	No. of observations	Anti-HBs Positive	Univariate analysis		Multivariable analysis	
		*n* (%)	*OR* (*95% CI*)	*P*-value	a*OR* (*95% CI*)	*P*-value
Maternal age (years)						
18–24	280	272 (97.1)	1.0 (0.4–2.2)	0.8		
25–34	2992	2893 (96.7)	0.8 (0.5–1.3)			
35–45	840	816 (97.1)				
Maternal educational level						
Junior middle school or below	1382	1341 (97.0)	1.1 (0.8–1.6)	0.6		
High Middle school or above	2730	2640 (96.7)	1			
Maternal residence rural / urban						
Rural	2818	2731 (96.9)	1.1 (0.8–1.6)	0.6		
Urban	1294	1250 (96.6)	1			
Maternal parity						
1	1732	1679 (96.9)	1.1 (0.8–1.5)	0.7		
> 1	2380	2302 (96.7)	1			
Maternal HBeAg status during this pregnancy						
Positive	1221	1155 (94.6)	0.4 (0.3–0.6)	< 0.001^#^	0.4 (0.3–0.6)	< 0.001
Negative	2740	2681 (97.8)	1		1	
Unknown	151	145 (96.0)	0.5 (0.2–1.3)		0.6 (0.2–1.3)	0.2
Maternal antiviral treatment during this pregnancy						
Yes	392	377 (96.2)	0.8 (0.5–1.4)	0.5		
No	3720	3604 (96.9)	1			
Level of delivery hospital						
Prefectural and above	1518	1463 (96.4)	0.8 (0.6–1.1)	0.2		
County level and below	2594	2518 (97.1)	1			
Mode of delivery						
Vaginal delivery	2349	2271 (96.7)	0.9 (0.6–1.3)	0.6		
Caesarean section	1763	1710 (97.0)	1			
Child gender						
Male	2185	2118 (96.9)	1.1 (0.8–1.5)	0.6		
Female	1927	1863 (96.7)	1			
Gestational age (weeks)						
< 37	236	225 (95.3)	0.7 (0.3–1.2)	0.2		
37–43	3876	3756 (96.9)	1			
Child birth weight (g)						
< 2500	131	122 (93.1)	0.4 (0.2–0.9)	0.02^*^	0.4 (0.2–0.8)	0.01
2500–5400	3981	3859 (96.9)	1		1	
Type of HBvacc-BD						
10 μg yeast vaccine	3637	3520 (96.8)	1.3 (0.6–2.7)	0.4		
20 μg CHO vaccine	285	279 (97.9)	2.0 (0.7–6.0)			
10 μg CHO vaccine	190	182 (95.8)	1			
Interval between birth and HBvacc-BD (hours)						
Within 12	3579	3467 (96.9)	1	0.5^*^		
12–24	466	450 (96.6)	0.9 (0.5–1.5)			
> 24	67	64 (95.5)	0.7 (0.2-2.2)			
Administered HBIG						
Yes	4092	3961 (96.8)	1.0 (0.96–0.97)	1.0^*^		
No	20	20 (100.0)	1			
Interval between 3rd dose HBvacc and HBV serological marker test (months)						
< 7	1666	1635 (98.1)	2.2 (1.5–3.4)	< 0.001	2.2 (1.5–3.4)	< 0.001
7–18	2446	2346 (95.9)	1		1	

*HBV* hepatitis B virus, *HBsAg* hepatitis B surface antigen, *anti-HBs* hepatitis B surface antibody, *HBvacc* hepatitis B vaccine, *HBvacc-BD* hepatitis B vaccine birth dose, *HBIG* hepatitis B immunoglobulin, *LBW* low birth weight, *CHO* Chinese hamster ovary, #: trend, *: Fisher’s exact test, the factors significant at *P* < 0.1 in univariate analysis entered into the regression model

## Discussion

We found strong programme performance with regards to timely HBIG and HBvacc-BD coverage and a relatively low MTCT rate and high sero-protection rate compared with previous research conducted in China for 2006–2014 [6, 9, 10]. We found most HBV-infected children were born to mothers with both HBsAg and HBeAg positivity during the pregnancy. Our results show progress towards rates comparable to those reported by the United States Perinatal Hepatitis B Prevention Programs for 2007–2013 (1.1%) [17]. Our finding of strong PMTCT performance among children with HBIG is consistent with current knowledge about efficiency and effectiveness of PMTCT of HBV by combined HBIG and three-dose HBvacc [18, 19].

The 2018–2030 WHO Western Pacific Regional framework for triple elimination of MTCT of HIV, HBV and syphilis in Asia and the Pacific set a regional target of prevalence of HBsAg among children of no more than 0.1% by 2030 [3]. In order to achieve the elimination goal in China, with an estimated 6% HBsAg prevalence among pregnant women, the MTCT rate needs to remain lower than 2% [12]. Our findings imply the national PMTCT programme for HBV is on track to achieve the elimination target by 2030 if efforts continue to sustain the progress achieved thus far and to tackle barriers for timely service access.

We demonstrated that the timing of HBvacc-BD was associated with risk of infection in children, with lower MTCT rates among children administered the HBvacc within 12 h of birth. Our findings are consistent with WHO recommendation that infants should receive HBvacc-BD as soon as possible from the time of and within 24 h of birth. The American Academy of Pediatrics endorses the recommendation to administer both HBvacc and HBIG within 12 h of birth, regardless of any maternal antenatal treatment with antiviral medication [20]. Our results provide additional evidence to verify the importance of timely HBvacc-BD and demonstrate a need to update the current national guidelines recommendation to a requirement for vaccination within 12 h of birth [8].

Our findings confirm the high risk of MTCT among children born to HBsAg- and HBeAg-positive mothers. HBeAg positivity is linked with higher levels of HBV replication and associated with intrauterine transmission of HBV [21]. The provision of antiviral prophylaxis has been reported as an additional protective measure to HBIG and HBvacc for children born to mothers with high HBV DNA level [22–24]. Our findings may be consistent with previous studies that showed a reduced MTCT risk associated with antiviral treatment for pregnant women with therapeutic indications [22–24]. However, the proportion of the pregnant women with HBV DNA test results who also received antiviral treatment in our study was fewer than 8%, possibly hindering the interpretation of these results.

We found the level of hospital associated with MTCT risk. We consider reflective of variances in the management of vaccination procedures and understanding of hepatitis B vaccination contraindications among health care providers at different levels of hospitals [25, 26]. The results indicate the need for further studies on quality of care with regards to HBIG and HBvacc immunization and their association with facility-level factors.

We found maternal HBeAg positivity was associated with lower sero-protection rates. This implies that children born to mothers who were both HBsAg and HBeAg positive are at a higher risk for either perinatal HBV infection or failure to obtain sero-protection from immunoprophylaxis. Children without sero-protection from anti-HBs may be at higher risk of infection from their HBsAg-positive mothers or other family members [27]. We found a small number of mothers with an unknown HBeAg status, despite screening being required by the national action plan [13], and ever few with a recorded HBV DNA test during pregnancy. Hence, it is important to standardize the provision of supplementary tests to HBsAg-positive pregnant women to identify those with HBeAg-positivity or high HBV DNA levels to provide appropriate medical care and follow-up services to this groups of mothers and their children.

Our findings of significantly lower sero-protection among children with LBW may be explained by: lower responsiveness of LBW infants to HBvacc [28] and/or delayed administration of HBvacc-BD (> 24 h of birth) for preterm birth or LBW [29, 30]. WHO recommends that infants born less than 2000 g should be given HBvacc-BD, but that the dose should not count as part of the primary three-dose HBvacc [31]. The US guidelines recommend that infants of all birth weights born to HBsAg-positive mothers receive both the HBvacc and HBIG within 12 h of birth [20]. In China, the 2016 National EPI guidelines similarly require HBvacc-BD regardless of birth weight for HBV-exposed infants, followed by the three-dose HBvacc series [8]. Our findings may point to the need for future studies on adherence to the guidelines for the timely provision of Hepatitis B vaccination for low birth weight infants born to HBsAg-positive mothers among healthcare providers.

### Limitations

Our research has several potential limitations. First, due to resource constraints, we undertook data collection over a limited period of time. As a result, about 60% of the HBV-exposed children received HBV serologic testing over seven months after their third dose of HBvacc, as opposed to the recommended window of one to two months during which anti-HBs levels are at their highest [9, 32]. In the absence of more stringent inclusion criteria, we may have estimated a lower sero-protection rate. Second, as we utilized convenience sampling, our study sample does not allow for generalization of results nation-wide. Therefore, our results may reflect selection bias for areas where the programme has been more effectively implemented. Hence, further studies are needed to understand the MTCT and sero-protection rates in other geographical regions of China, particularly in areas with poor programme performance. Third, insufficient availability of HBV DNA test results for mothers limited our ability to investigate any association between and HBV DNA levels and MTCT and sero-protection rates. However, as the majority of our data were extracted from hospital records or information systems, we were able to reduce potential recall bias.

## Conclusions

This study found an MTCT rate among children born to HBsAg-positive mothers lower than 2%, which reflects the effectiveness and efficiency of the national programme. These results are encouraging with respect to the 2030 elimination goal [3]. Hence, we recommend continuing to strengthen current PMTCT intervention strategies to ensure immunoprophylaxis including timely HBvacc series and HBIG. It is crucial to emphasize early HBvacc-BD (within 12 h at birth, as soon as possible) for HBV-exposed infants, which may be achieved by an amendment to the current EPI guidelines. In the context of high coverage of post-exposure immunoprophylaxis interventions, infants born to HBeAg-positive mothers still face a higher risk of HBV infection and a lack of sero-protection. In order to eliminate HBV MTCT, it is necessary to identify all HBeAg-positive mothers during pregnancy through providing all HBsAg positive pregnant women with HBeAg test and providing identified mother-child pairs with appropriate medical care and follow-up. Finally, we recommend future studies on the implementation of HBvacc-BD among preterm or LBW HBV-exposed infants as well as studies on health facility-level factors and their association to MTCT and sero-protection rates.

## Additional file

Additional file 1: Multilingual abstracts in the five official working languages of the United Nations. (PDF 228 kb)

## Data Availability

Data is available upon reasonable request to the corresponding author.

## Abbreviations

anti-HBs: HBV surface antibody
a*OR*: Adjusted odds ratio
CDC: Centres for Diseases Control and Prevention
CHO: Chinese hamster ovary cells
*CI*: Confidence interval
ELISA: Enzyme linked immunosorbent assay
EPI: Expanded Programme on Immunization
HBeAg: Hepatitis B e antigen
HBIG: Hepatitis B immunoglobulin
HBsAg: Hepatitis B surface antigen
HBV: Hepatitis B virus
HBvacc: Hepatitis B vaccine
HBvacc-BD: Hepatitis B vaccine birth dose
HIV: Human immunodeficiency virus
IU: International unit
LBW: Low birth weight
MTCT: Mother-to-child transmission
*OR*: Odds ratio
PMTCT: Prevention of mother-to-child transmission
*SD*: Standard deviation
US: The United States
WHO: World Health Organization
