# Supplementary material for: Outcomes of the national programme on prevention of mother-to-child transmission of hepatitis B virus in China, 2016–2017
Source: Infect Dis Poverty. 2019 Aug 5;8:65. doi: 10.1186/s40249-019-0576-y (PMC6681489; doi:10.1186/s40249-019-0576-y)
Supplement: Supplementary file 1 — Multilingual abstracts in the five official working languages of the United Nations. (PDF 228 kb) [file 40249_2019_576_MOESM1_ESM.pdf]

Translation of the abstract into the five official working languages of the United Nations

نتائج البرنامج الوطني المعني بالوقاية من انتقال فيروس التهاب الكبد الوبائي B من الأم إلى الطفل في الصين، 2016-2017

يا بينغ تشياو، مين سوياو سونغ، شياو يان وانغ، تشن لي، يان لين لي، لي شيا دو، تشيان وانغ، أترينا هان، قوه مين تشانغ، شياو نا هوانغ، يو نينغ يانغ،
شي جين، آي لينغ وانغ

نبذة
معلومات أساسية: بالإضافة إلى توفير السلسلة المجانية من لقاح فيروس الكبد الوبائي (HBvacc) لجميع الأطفال في الصين منذ عام 2005، بدأ البرنامج الوطني المعني بالوقاية من انتقال فيروس التهاب الكبد الوبائي B من الأم إلى الطفل (PMTCT) بتوفير الغلوبولين المناعي المجاني لالتهاب الكبد الوبائي B لجميع الرضع الذين يولدون من أمهات مصابات بفيروس التهاب الكبد الوبائي B في عام 2010. ومع ذلك، فقد قيمت دراسات قليلة فعالية برنامج PMTCT. لذلك، فإننا نهدف إلى استقصاء نتائج البرنامج وتحديد ما يتصل به من عوامل.
المنهج: باستخدام تصميم لدراسة مقطعية مستعرضة، جمعنا بيانات عن 4112 زوج من الأمهات المصابات بفيروس التهاب الكبد الوبائي B وأطفالهن الذين تتراوح أعمارهم ما بين 7 و22 شهرًا في أربع مقاطعات تمثيلية من خلال المقابلات واستعراض السجلات الطبية. اختبرنا وجود مولدات الأجسام المضادة السطحية التي تؤكد الإصابة بالتهاب الكبد الوبائي B واختصارها (HBsAg) والأجسام المضادة السطحية التي تؤكد مناعة الجسم من التهاب الكبد الوبائي B واختصارها (anti-HBs) بين الأطفال عن طريق مقايسة الامتصاص المناعي المرتبط بالأنزيمات في مختبرات محددة بمستشفيات الأم والطفل. استخدمنا الانحدار اللوجستي لتحليل العوامل المرتبطة بوجود مولدات الأجسام المضادة السطحية (HBsAG) ووجود الأجسام المضادة السطحية (anti-HBs) لدى الأطفال.
النتائج: أظهرت نتائج الاختبار وجود مولدات الأجسام المضادة السطحية (HBsAg) لدى خمسة وثلاثين طفلاً، مما يشير إلى أن نسبة انتقال الفيروس من الأم إلى الطفل كانت 0.9٪ (1.1–0.6) وكانت نسبة وجود الأجسام المضادة السطحية (anti-HBs) قيمتها 96.8% (96.3-97.4 ٪). الأطفال الذين جرى تطعيمهم بلقاح HBvacc في الساعات الأولى من ولادتهم، ما بين12 و24 ساعة، ازداد ترجيح تعرضهم للإصابة بالفيروس 2.9 ضعف احتمال إصابة الأطفال الذين جرى تطعيمهم في أقل من 12 ساعة من وقت ولادتهم (نسبة الاحتمالات المعدَّلة [aOR] = 2.9 95% مجال الثقة [CI]: 1.4–6.3, P = 0.01). وارتبط وجود مولدات الأجسام المضادة (HBeAg) لدى الأمهات بارتفاع نسبة انتقال الفيروس من الأم إلى الطفل (aOR = 79.1)95%CI: 10.8–580.2(, P < 0.001) وانخفاض نسبة وجود الأجسام السطحية المضادة (aOR = 0.4)95%CI: 0.3–0.6(, P < 0.001). والرضع المنخفضو الوزن (LBW) كانت نسبة ترجيح وجود الأجسام المضادة السطحية (anti-HBs) لديهم 60٪ أقل من الرضع الذين كان وزنهم طبيعيًا عند الولادة (a OR = 0.4 95٪ CI : 0.2–0.8(، P = 0.01).
الاستنتاجات : انخفضت نسبة انتقال الفيروس من الأم إلى الطفل MTCT مقارنةً بالنسبة المحددة في هدف القضاء على التهاب الكبد الوبائي بحلول عام 2030 الذي وضعته منظمة الصحة العالمية، مما يشير إلى أن البرنامج يسير على الطريق الصحيح لتحقيق هذا الهدف. نظرًا لأن التبكير في جرعة لقاح فيروس الالتهاب الكبدي الوبائي للمولودين (HBvcc-BD) كان مرتبطًا بانخفاض نسبة انتقال العدوى من الأم إلى الطفل MTCT، فإننا نقترح أن يعمل برنامج PMTCT مع البرنامج الموسع للتحصين (EPI) لتعديل التوصية الحالية بشأن التبكير في التطعيم بلقاح HBvcc-BD كي تصبح مطلبًا. وتشير النتيجة التي خلصنا إليها أن انخفاض وزن الرضع LBW كان مرتبطًا بنسبة أقل من وجود الأجسام المضادة (anti-HBs) إلى الحاجة إلى مزيد من الدراسات لفهم العوامل المرتبطة بهذه المخاطر وفرص تعزيز البرنامج. ويجب أن يضمن البرنامج توفير اختبار أساسي لتحديد الأمهات المصابات بفيروس الكبد الوبائي HBeAg وأطفالهن، وتزويدهم بما يلائمهم من رعاية طبية ومتابعة.

Translated from English version into Arabic by Aalya Al-Beeshi, Revised by Shaima'a al-Fahel, through

2016-2017 年中国预防乙肝母婴传播干预效果

Ya-Ping Qiao, Min SuYao Song, Xiao-Yan Wang, Zhen Li, Yan-Lin Li, Li-Xia Dou, Qian Wang, atrina Hann, Guo-Min Zhang, Xiao-Na Huang, Yu-Ning Yang,
Xi Jin, Ai-Ling Wang

摘要
引言：自 2005 年起，中国向所有婴儿提供免费的乙肝疫苗接种。在此基础上，全国预防乙肝母婴传播项目自 2010 年起向所有乙肝表面抗原（hepatitis B surface-antigen，HBsAg）阳性母亲所生新生儿提供免费的乙肝免疫球蛋白接种。然而，评价预防乙肝母婴传播项目效果研究很少。为了解开展预防母婴传播项目后的干预效果，明确相关影响因素，特开展本研究。
方法：本研究为横断面调查，通过问卷调查和医疗资料查阅收集了 4 个代表省的 4112 对 HBsAg 阳性母亲及所生 7-22 个月龄儿童的相关数据，同时在指定的妇幼保健院实验室采用酶联免疫吸附试验方法检测儿童血清中 HBsAg 和乙肝表面抗体（抗-HBs）。采用 logistic 回归模型分析儿童 HBsAg 或抗-HBs 阳性的影响因素。
结果：本研究中 35 名儿童 HBsAg 阳性，母婴传播率为 0.9%（0.6%-1.1%），抗-HBs 阳性率为 96.8%（96.3%-97.4%）。出生后 12-24 小时内接种首针乙肝疫苗的儿童发生乙肝感染的可能性是出生后 12 小时内接种乙肝疫苗的儿童的 2.9 倍（aOR=2.9, 95%CI: 1.4-6.3, P=0.01）。妊娠期母亲乙肝 e 抗原（hepatitis B e-antigen，HBeAg）阳性与较高的母婴传播率相关（aOR=79.1, 95%CI: 10.8-580.2, P<0.001）和较低的抗-HBs 阳性率相关（aOR=0.4, 95%CI: 0.3-0.6, P<0.001）。低出生体重儿的抗-HBs 阳性率较正常出生体重儿低 60%（aOR=0.4, 95%CI: 0.2-0.8, P=0.01）。
结论：本研究中乙肝母婴传播率低于 2030 年世界卫生组织消除乙肝母婴传播目标，提示全国预防母婴传播项目实施将有望实现这一目标。出生后及早接种首针乙肝疫苗与较低的母婴传播率相关，建议将出生后及早接种首针乙肝疫苗纳入全国预防母婴传播工作和扩大国家免疫规划相关要求中。低出生体重与较低的抗-HBs 阳性相关，提示需要开展进一步研究了解相关危险因素及改进机会。建议在全国预防母婴传播工作实施过程中，确保提供必要的检测以发现 HBeAg 阳性母亲及所生婴儿，并为其提供适宜的医疗保健和随访服务。

Translated from English version into Chinese by Ya-Ping Qiao

Résultats du programme national de prévention de la transmission mère-enfant du virus de l'hépatite B, en Chine, 2016-2017

Ya-Ping Qiao, Min SuYao Song, Xiao-Yan Wang, Zhen Li, Yan-Lin Li, Li-Xia Dou, Qian Wang, atrina Hann, Guo-Min Zhang, Xiao-Na Huang, Yu-Ning Yang,
Xi Jin, Ai-Ling Wang

Résumé

Contexte : En plus d'offrir gratuitement, depuis 2005, la série de vaccins contre l'hépatite B (HBvacc) à tous les nourrissons de Chine, le programme national de prévention de la transmission mère-enfant (PTME) du virus de l'hépatite B (VHB) a commencé d'offrir gratuitement, en 2010, l'immunoglobuline de l'hépatite B à tous les nouveau-nés de mères séropositives pour l'antigène de surface de l'hépatite B (AgHBs). Cependant, peu d'études ont évalué l'efficacité du programme de PTME. Notre objectif consistait donc à investiguer sur les résultats du programme et à identifier les facteurs associés.

Méthode : Par le biais d'une étude transversale, nous avons recueilli des données, grâce à des entretiens et à un examen des dossiers médicaux, sur 4 112 couples formés de mères séropositives pour le AgHBs et de leur enfant âgé de 7 à 22 mois, dans quatre provinces représentatives. Les enfants ont été soumis à un test, pour le AgHBs et l'anticorps de surface de l'hépatite B (anti-HBs), utilisant la méthode immuno-enzymatique ELISA dans les laboratoires désignés d'hôpitaux offrant des soins maternels et infantiles. Nous avons eu recours à la régression logistique pour analyser les facteurs associés à un résultat positif pour les AgHBs et anti-HBs chez les enfants.

Résultats: Trente-cinq enfants étaient séropositifs pour le AgHBs, ce qui indique un taux de transmission mère-enfant (TME) de 0,9 % (0,6 – 1,1 %). Le taux d'enfants positifs pour l'anti-HBs était de 96,8 % (96,3 - 97,4 %). Les enfants recevant le vaccin contre l'hépatite B (HBvacc) dans les 12 à 24 heures suivant la naissance étaient 2,9 fois plus susceptibles d'être infectés par rapport à ceux vaccinés moins de 12 heures après la naissance (odds ratio ajusté [a*OR*] = 2,9, intervalle de confiance de 95% [*IC*] : 1,4 – 6,3, *P* = 0,01). La séropositivité des mères pour l'antigène e de l'hépatite B (AgHBe) était associée à un taux supérieur de TME (a*OR* = 79,1 (*IC* 95 % : 10,8 – 580,2), *P* < 0,001) et à un taux de résultats positifs pour l'anti-HBs moindre (a*OR* = 0,4 (*IC* 95 % : 0,3 – 0,6), *P* < 0,001). Les enfants présentant un faible poids de naissance (FPN) avaient 60 % de chances en moins d'avoir un résultat positif pour l'anti-HBs par rapport aux enfants présentant un poids normal à la naissance (a*OR* = 0,4 (*IC* 95% : 0,2 – 0,8), *P* = 0,01).

Conclusions : Le taux de TME était moindre par rapport à l'objectif d'élimination établi par l'OMS pour 2030, ce qui suppose que le programme est en voie d'atteindre cette cible. Étant donné que la dose de vaccin contre l'hépatite B administrée à la naissance (HBvcc-BD) était associée à un taux moindre de TME lorsqu'elle était reçue précocement, nous suggérons que le programme de PTME collabore avec le programme élargi de vaccination (PEV) pour transformer en obligation la recommandation actuelle conseillant une administration précoce du HBvcc-BD. L'association entre FPN et moindre chances d'être positif pour l'anti-HBs, démontrée par la présente étude, indique le besoin de réaliser des études complémentaires afin de comprendre les facteurs associés à ces risques et ces chances en vue de renforcer le programme. Il faut que le programme veille à offrir un test, d'une importance essentielle, permettant d'identifier les mères séropositives pour l'AgHBe et leurs nourrissons, et qu'il leur fournisse les soins médicaux et le suivi approprié.

Translated from English version into French by Cendrine Strevens, Revised by Joelle Bescond, through

TRANSLATORS
WITHOUT BORDERS

Результаты национальной программы по профилактике передачи вируса гепатита В от матери ребенку в 2016–2017 гг. в Китае

Я-Пин Цяо, Минь СуЯо Сун, Сяо-Янь Ван, Чжэн Ли, Янь-Линь Ли, Ли-Ся Доу, Сянь Ван, Атрина Ханн, Го-Минь Чжан, Сяо-На Хуан, Юй-Нин Ян,
Си Цзинь, Ай-Лин Ван

Аннотация

Предпосылки: Помимо предоставления бесплатных серий вакцин против гепатита В (HBvacc) всем новорожденным в Китае с 2005 года в 2010 г. по национальной программе профилактики передачи от матери ребенку (ППМР) вируса гепатита В (HBV) родившимся у матерей с положительным поверхностным антигеном (HBsAg) гепатита В начали вводить бесплатный иммуноглобулин против гепатита В. Тем не менее, эффективность программы ППМР оценивали мало исследований. Поэтому мы стремились исследовать результаты программы и определить связанные с этим факторы.

.**Метод:** в перекрестном исследовании мы собрали данные о 4112 парах HBsAg-позитивных матерей и их детей в возрасте 7–22 месяцев в четырех репрезентативных провинциях посредством опросов и обзора медицинских карт. Мы проверили поверхностные антитела HBsAg и гепатита В (анти-HBs) у детей с помощью иммуноферментного анализа в специализированных лабораториях для матерей и детей. Мы использовали логистическую регрессию для анализа факторов, связанных с детским HBsAg и анти-HBs-позитивностью.

Результаты: тридцать пять детей были HBsAg-положительными, что указывает на то, что уровень передачи от матери ребенку (MTCT) составил 0,9% (0,6–1,1%). Положительный уровень анти-HBs составил 96,8% (96,3–97,4%). У детей, получавших HBvacc в возрасте 12–24 часов после рождения, вероятность инфицирования в 2,9 раза выше, чем у детей, вакцинированных менее чем за 12 часов (скорректированное отношение шансов [a *OR*] = 2,9, доверительный интервал 95% [*CI*]: 1,4–6,3, *P* = 0.01) Позитивность е-антигена материнского гепатита В (HBeAg) была связана с более высоким уровнем MTCT (a*OR* = 79,1 (95%*CI*: 10,8–580,2), *P* < 0,001) и пониженным уровнем анти-HB (a*OR* = 0,4 (95%*CI*: 0,3–0,6), *P* < 0,001). Дети с низкой массой тела при рождении (LBW) были на 60% менее склонны к HB-позитивности, чем дети с нормальной массой тела при рождении (a*OR* = 0,4 (95% *CI*: 0,2–0,8), *P* = 0,01).

Выводы: Показатель ППМР был ниже цели ликвидации ВОЗ 2030 г., что означает, что программа находится на пути к достижению своей цели. Поскольку более ранняя доза HBvacc при рождении (HBvcc-BD) была связана с более низким уровнем MTCT, то мы предлагаем, чтобы программа PMTCT работала с Расширенной программой иммунизации (EPI) для того, чтобы изменить текущую рекомендацию для раннего HBvcc-BD в соответствии с требуемым. Наш вывод о том, что LBW связан с более низким положительным анти-HBs, указывает на необходимость дальнейших исследований для понимания факторов, связанных с этими рисками, и возможностей для усиления программы. Программа должна обеспечить предоставление необходимого теста для выявления HBeAg-позитивных матерей

и их детей и предоставления им надлежащей медицинской помощи и последующего наблюдения.

Translated from English version into Russian by Kenul Aliyeva-Aqarzayeva, Revised by Alexander Somin, through

TRANSLATORS
WITHOUT BORDERS

Resultados del Programa Nacional para la Prevención de la transmisión madre a hijo del virus de la hepatitis B en China, 2016-2017

Ya-Ping Qiao, Min SuYao Song, Xiao-Yan Wang, Zhen Li, Yan-Lin Li, Li-Xia Dou, Qian Wang, atrina Hann, Guo-Min Zhang, Xiao-Na Huang, Yu-Ning Yang,
Xi Jin, Ai-Ling Wang

Resumen

Antecedentes: además de proporcionar de manera gratuita una serie de vacunas contra la hepatitis B (vacHB) a todos los niños en China desde 205, el Programa Nacional de Prevención de la transmisión madre a hijo (PTMH) del virus de la hepatitis B (VHB) comenzó a proporcionar sin coste alguno inmunoglobulina anti-hepatitis B para todos los recién nacidos de madres con pruebas positivas para el antígeno de superficie de la hepatitis B (HBsAg) en 2010. Sin embargo, pocos estudios han evaluado la eficacia del programa de PTMH. Por consiguiente, tratamos de investigar los resultados del programa e identificar los factores relacionados.

Método: utilizando un diseño de estudio transversal, recopilamos datos de 4 112 parejas de madres con pruebas positivas para el HBsAG y sus hijos de 7-22 meses de edad en cuatro provincias representativas a través de entrevistas y de la revisión del historial médico. Realizamos pruebas de detección del HBsAg y del anticuerpo de superficie de la hepatitis B (anti-HB) en los niños por medio de un ensayo inmunoenzimático en laboratorios designados de hospitales materno-infantiles. Usamos una regresión logística para analizar los factores relacionados con la positividad de HBsAg y de anti-HB en los niños.

Resultados: treinta y cinco niños dieron positivo para HBsAg, lo cual indica que el índice de transmisión madre a hijo (TMH) fue del 0,9% (0,6% - 1,1%). El índice de resultados positivos para anti-TB fue del 96,8% (96,3% - 97,4%). Los niños que recibieron vacHB dentro de las primeras 12 a 24 horas después del nacimiento fueron 2,9 veces más vulnerables a la infección que los que fueron vacunados en menos de 12 horas (razón de posibilidades ajustada [*OR*a] = 2,9, intervalo de confianza del 95% [*IC*]: 1,4-6,3, *P* = 0,01) El resultado positivo del antígeno e de hepatitis B (HBeAg) en las madres estuvo relacionado con un mayor índice de TMH (*OR*a = 79,1 (*IC* del 95%: 10,8–580,2), *P* < 0,001) y un menor índice positivo de anti-HB (*OR*a = 0,4 (*IC* del 95%: 0,3–0,6), *P* < 0,001). Los niños con bajo peso al nacer (BPN) tuvieron un 60% menos de probabilidades de dar positivo para anti-TB que aquellos con peso normal al nacer (*OR*a = 0,4 (*IC* del 95%: 0,2–0,8), *P* = 0,01).

Conclusiones: el índice de TMH fue menor que el objetivo de la OMS para la eliminación para 2030, lo cual implica que el programa está en vías de alcanzar este objetivo. Dado que la administración más temprana de una dosis de la vacHB al nacer estuvo relacionada con un menor índice de TMH, recomendamos que el programa de PTMH colabore con el Programa Ampliado de

Inmunización (PAI) para modificar la recomendación actual de una dosis temprana de vacHB al nacer a fin de convertirla en un requisito. Nuestro hallazgo de que el BPN estuvo relacionado con menos resultados positivos para anti-TB señala la necesidad de realizar más estudios a fin de comprender los factores relacionados con estos riesgos y oportunidades para el fortalecimiento del programa. El programa debe garantizar que se realicen las pruebas esenciales para identificar a las madres con pruebas positivas para HBeAg y sus hijos, así como proporcionarles la atención médica y el seguimiento adecuados.

Translated from English version into Spanish by Mayra León, Revised by Maria Gracia Zavarse, through

TRANSLATORS
WITHOUT BORDERS
